# Functional Connection between Rad51 and PML in Homology-Directed Repair

**DOI:** 10.1371/journal.pone.0025814

**Published:** 2011-10-05

**Authors:** Sergei Boichuk, Liang Hu, Kathleen Makielski, Pier Paolo Pandolfi, Ole V. Gjoerup

**Affiliations:** 1 Cancer Virology Program, University of Pittsburgh Cancer Institute, Pittsburgh, Pennsylvania, United States of America; 2 Department of Medicine, Beth Israel Deaconess Cancer Center, Harvard Medical School, Boston, Massachusetts, United States of America; The University of Hong Kong, China

## Abstract

The promyelocytic leukemia protein (PML) is a tumor suppressor critical for formation of nuclear bodies (NBs) performing important functions in transcription, apoptosis, DNA repair and antiviral responses. Earlier studies demonstrated that simian virus 40 (SV40) initiates replication near PML NBs. Here we show that PML knockdown inhibits viral replication *in vivo*, thus indicating a positive role of PML early in infection. SV40 large T antigen (LT) induces DNA damage and, consequently, nuclear foci of the key homologous recombination repair protein Rad51 that colocalize with PML. PML depletion abrogates LT-induced Rad51 foci. LT may target PML NBs to gain access to DNA repair factors like Rad51 that are required for viral replication. We have used the SV40 model to gain insight to DNA repair events involving PML. Strikingly, even in normal cells devoid of viral oncoproteins, PML is found to be instrumental for foci of Rad51, Mre11 and BRCA1, as well as homology-directed repair after double-strand break (DSB) induction. Following LT expression or external DNA damage, PML associates with Rad51. PML depletion also causes a loss of RPA foci following γ-irradiation, suggesting that PML is required for processing of DSBs. Immunofluorescent detection of incorporated BrdU without prior denaturation indicates a failure to generate ssDNA foci in PML knockdown cells upon γ-irradiation. Consistent with the lack of RPA and BrdU foci, γ-irradiation fails to induce Chk1 activation, when PML is depleted. Taken together, we have discovered a novel functional connection between PML and the homologous recombination-mediated repair machinery, which might contribute to PML tumor suppressor activity.

## Introduction

Nuclear substructures, known as promyelocytic leukemia (PML) nuclear bodies (NBs), PML oncogenic domains (PODs) or nuclear domain 10 (ND10), are associated with nuclear matrix and play important roles in diverse cellular processes such as transcription, chromatin dynamics, oncogenesis, posttranslational modifications, apoptosis, p53 regulation, senescence, DNA damage repair and antiviral responses [1], [2], [3], [4]. PML is the nucleating component of PML NBs, essential for their formation, and loss of PML results in dispersal of the other components residing at PML NBs. There is known to be a rapid exchange between the PML NBs and the nucleoplasm [1].

At least seven PML isoforms exist due to alternative splicing of the C-terminal exons, thus generating different protein interaction domains [5]. There are constitutive components of PML NBs such as PML, DAXX and Sp100 but also a large number (>50) of proteins that can transiently associate with PML NBs, for example p53 and p300/CBP [1]. PML, as well as several other PML NB components, are modified by small ubiquitin-like modifier (SUMO) and for PML this is essential for formation and maintenance of the PML NBs [6], [7].

PML is a tumor suppressor protein as first realized in acute promyelocytic leukemia (APL), where a chromosomal translocation creates a fusion protein with the retinoic acid receptor α, resulting in loss of PML function. Moreover, PML expression is lost in a number of solid tumors, the loss being correlated with tumor progression [8]. PML knockout mice are developmentally normal and do not succumb to spontaneous tumors but are more susceptible to chemical carcinogens [9].

Three hypotheses exist regarding the general function of PML NBs [1], [2]. One of these posits that PML NBs serve as nuclear depots for various factors that are then released in a precise spatiotemporal manner following DNA damage or stress. Another model envisions that PML NBs are key sites orchestrating posttranslational modification, thus acting as “catalytic surfaces”. Finally, PML NBs could also be defined sites for a specific nuclear function like transcription, replication or chromatin remodeling. Relevant for our current studies, PML NBs were previously implicated in DNA repair pathways and genomic instability, although mechanisms remain incompletely understood [3], [10], [11], [12], [13], [14].

Viruses have evolved a complex relationship with PML NBs and the debate is ongoing as to whether they exert a positive or a negative effect on the viral life cycle [2], [4], [15]. On one hand, genomes from different virus families deposit their incoming genomes adjacent to, or at the periphery of PML NBs at early time points after infection (herpes simplex virus 1 (HSV-1), human cytomegalovirus (HCMV), adenovirus, SV40) [16], [17], [18], [19]. Conversely, viruses such as HSV-1 and HCMV target PML NBs for inactivation, suggesting that they mediate an antiviral function [2], [4], [15]. SV40 clearly differs from these viruses by initiating its replication at specific nuclear sites near PML NBs, without obvious inactivation of these structures having been observed [16], [19].

Here we report on the interplay between SV40 and PML NBs. Interestingly, viral replication is significantly impaired following siRNA-mediated PML depletion. As a possible mechanism, we propose that LT targets PML NBs to gain access to repair factors like Rad51 that are needed for viral genome repair. We previously demonstrated that LT induces DNA damage, and consequently, foci of the Rad51 recombinase [20]. This suggests an induction of the homologous recombination (HR) repair machinery, analogous to what happens in normal cells after ionizing radiation (IR) generates double-strand breaks (DSBs). Furthermore, LT colocalizes with PML and Rad51, the latter being required for SV40 origin-dependent replication [20]. Here we show that PML is required for proper localization of Rad51 in foci generated after LT expression or DSB generation in normal cells. Moreover, efficient homology-directed recombinational repair and Chk1 activation depends on PML in cells devoid of viral oncoproteins. Based on this novel use of SV40 as a model system for basic cellular DNA repair events, we have identified a functional role of PML in HR repair following DNA damage.

## Results

### PML depletion significantly attenuates in vivo SV40 origin-dependent replication

**Figure 1:**
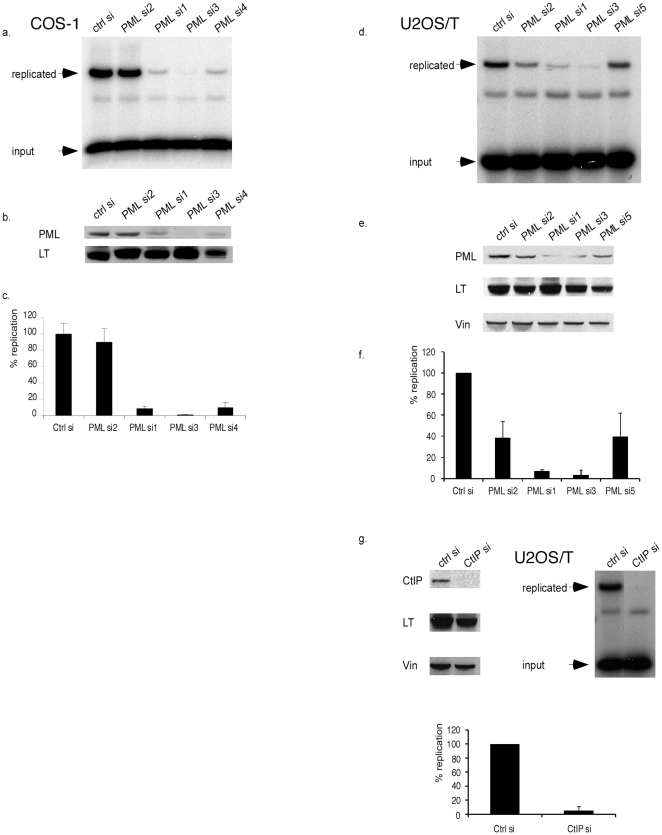
PML or CtIP knockdown attenuates SV40 origin-dependent replication. **A**. Four independent PML siRNAs, or a control (ctrl), were transfected into COS-1 cells for 48 h, followed by transfection of a SV40 origin-containing plasmid for 24 h. Low molecular weight DNA (Hirt) was extracted, digested with HincII/DpnI, and replicated DNA detected by Southern blotting using an origin-specific probe. Input DNA is also indicated. **B**. Lysates, from parallel dishes used for the replication assay, were analyzed by Western blotting for PML and LT expression. Si2 did not knock down PML, thus serving as a control. **C**. Graph depicting average and standard deviation (SD) from three independent replication assays. **D**. Replication assay in U2OS/T cells. Si5 is included [18]. **E**. Lysates of transfected cells were analyzed for PML, LT and vinculin (vin) expression by Western blotting. Vinculin was included as a loading control. **F**. The graph depicts average and SD from three independent replication assays in U2OS/T. **G**. Western blotting confirms knockdown of CtIP, as well as even LT and vinculin expression. The replication assay demonstrates reduced replication upon CtIP silencing. The graph illustrates the average and SD of three experiments.

It was previously suggested that SV40 replication preferentially occurs at specific sub-nuclear sites near PML NBs, but this has not been substantiated [16], [19]. To determine *in vivo* viral replication proficiency, we used COS-1 monkey kidney cells that constitutively express LT. Cells were depleted of endogenous PML by transfection with either of three different and target-specific siRNAs for 48 h (si1, si3 and si4), followed by transfection of an SV40 origin construct for another 24 h [20]. Viral replication was assayed by determining acquired resistance to DpnI digestion of isolated low molecular weight DNA using Southern blotting. When compared with the non-targeting siRNA, or an essentially non-functional PML siRNA (si2), it was evident that PML knockdown causes substantial reduction in viral replication (Fig. 1a). The degree of replication inhibition parallels the extent of PML knockdown as determined by Western blotting (Fig. 1b). As a control, each replication assay sample was shown to be comparable for LT expression by immunoblotting (Fig. 1b). Replication results from three experiments were quantitated and graphically depicted in Fig. 1c. An independent assay of viral replication in COS-1 based on a luciferase reporter construct with an SV40 origin produced very similar results (Fig. S1, [21]).

To corroborate and extend our conclusions with COS-1 cells, we repeated the experiment in human U2OS cells stably expressing LT (U2OS/T) (Fig. 1d). Similar results were obtained with si1 and si3 (si4 is monkey sequence specific), indicating that PML knockdown also impairs replication in human cells. A previous report had found no effect of PML knockdown on viral replication proficiency using real-time PCR in MCF-7 [18]. Using their siRNA (si5), we also found only a modest effect on viral replication, which is likely due to inefficient PML depletion (Fig. 1e). Taken together, whenever PML is significantly depleted, origin-dependent replication becomes attenuated. The graph depicts U2OS/T replication data (Fig. 1f).

Knockdown of either PML or Rad51 significantly impairs viral replication (Fig. 1a, Fig. 1d, [20]). To corroborate a potential link with HR repair, we examined CtIP, a factor required for processing of DSB ends by resection, which is a prerequisite for RPA and Rad51 loading [22], [23]. Depletion of CtIP with siRNA markedly reduces viral replication in U2OS/T, supporting the argument that HR repair is implicated (Fig. 1g). Western blotting indicates knockdown of CtIP, while maintaining LT expression (Fig. 1g).

### PML is required for enhanced Rad51 stability and spatial localization in foci

We previously showed that LT induces Rad51 foci [20]. Rad51 localization in DNA damage-induced foci is likely to reflect HR repair events [24], [25], [26], [27]. Moreover, LT localizes at, or near, Rad51 foci, together with PML [20]. We speculate that LT might target PML NBs to gain access to HR repair factors like Rad51, as it has been hypothesized [28].

**Figure 2:**
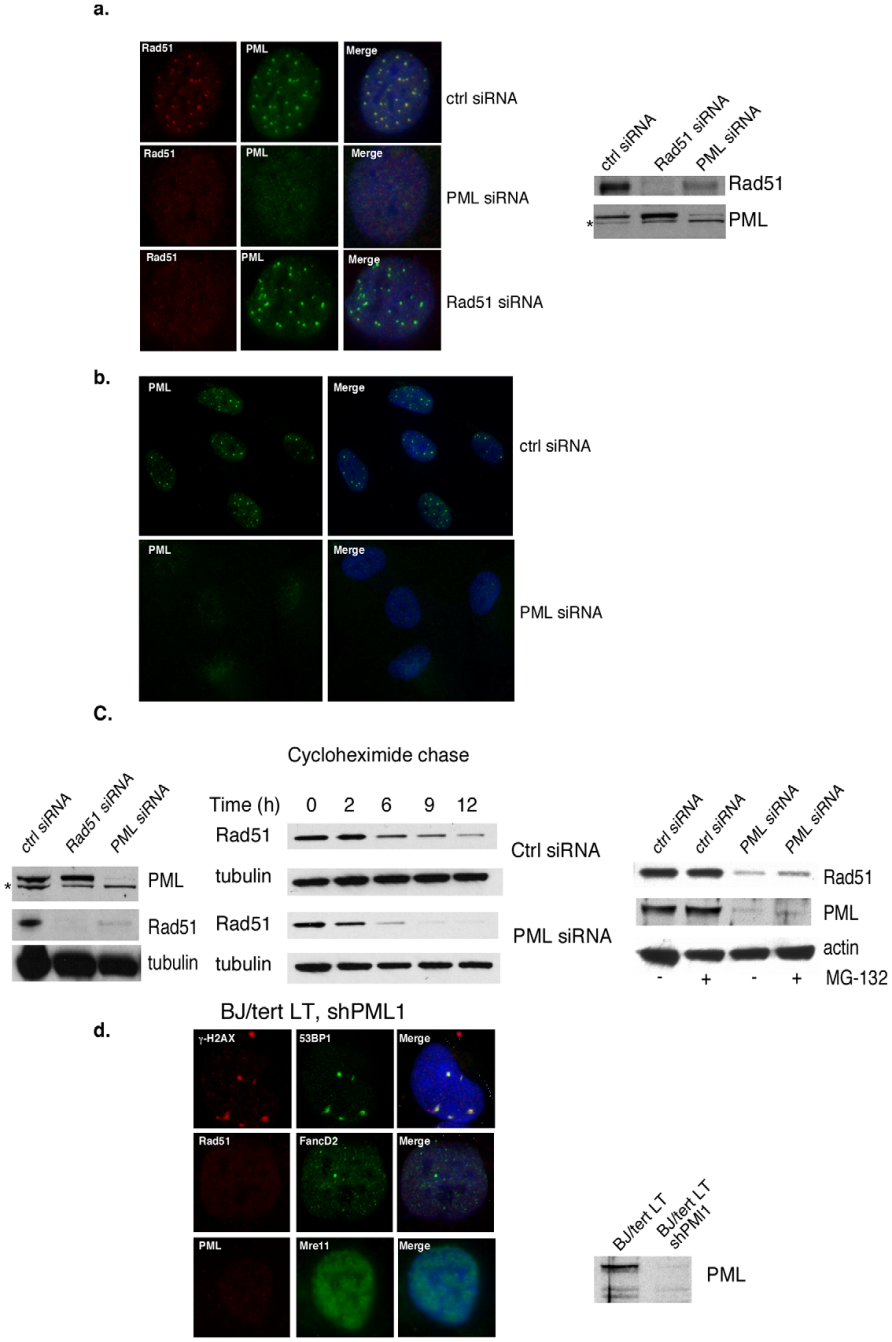
PML is required for regulation of Rad51 stability and focal accumulation upon LT expression. **A**. BJ/tert LT cells were transfected with control, PML or Rad51 siRNA for 48 h, followed by double immunofluorescence staining for Rad51 and PML. Images were also merged with DAPI staining to outline the nucleus. BJ/tert LT cells transfected with control, Rad51 or PML siRNA were also subjected to immunoblotting with PML and Rad51 antibodies. The * indicates a likely non-specific band. **B**. Immunofluorecence staining for PML was performed on BJ/tert LT cells after control or PML siRNA transfection. A wider field of cells is shown. **C**. U2OS cells transfected with control, Rad51 or PML siRNA were analyzed by Western blotting using PML, Rad51 and tubulin antibody. The half-life of Rad51 was determined by cycloheximide chase analysis. U2OS cells were transfected with control or PML siRNA for 48 h, followed by treatment with 10 µg/ml cycloheximide for 0, 2, 6, 9 or 12 h. Immunoblotting for Rad51 and tubulin was performed on samples from all time points. To investigate if proteasomal degradation occurs, cells were treated with MG-132 for 6 h. **D**. BJ/tert LT cells stably transduced with a PML shRNA (shPML1, [29]) were analyzed by immunofluorescence for γ-H2AX/53BP1, Rad51, PML, FancD2 and Mre11 foci. Knockdown efficiency was also determined by PML immunoblotting. Note that a different PML antibody was used from a. and c.

Therefore, we investigated by immunofluorescence if the Rad51 foci observed in BJ/tert cells stably expressing LT are dependent on PML. As shown in Fig. 2a, Rad51 foci are colocalized with PML in BJ/tert LT cells transfected with control siRNA [20], whereas transfection with PML siRNA causes loss of PML staining and faint, diffuse pan-nuclear accumulation of Rad51. A larger field of cells stained by immunofluorescence for PML confirms that siRNA-mediated knockdown is almost complete, with only rare, very faint PML dots (Fig. 2b). Interestingly, siRNA-mediated depletion of Rad51 does not affect the appearance of PML foci (Fig. 2a). Knockdown of Rad51 and PML was confirmed by immunoblotting, which reveals that PML siRNA also reduces Rad51 protein levels (Fig. 2a). Similarly to BJ/tert cells, PML knockdown in U2OS reduces Rad51 levels. A cycloheximide chase experiment indicates that the Rad51 half-life is shortened (Fig. 2c). The decreased stability of Rad51 does not appear to be due to ubiquitin-mediated proteasomal degradation, since treatment with the proteasome inhibitor MG-132 fails to significantly restore the levels of Rad51 (Fig. 2c).

PML was also stably depleted in BJ/tert LT with a retroviral vector encoding an shRNA corresponding to si1. This construct was previously used to stably silence PML expression [29], [30]. As shown in Fig. 2d, PML was almost completely depleted by Western blotting and immunofluorescence analysis. We previously demonstrated that LT induces multiple classes of DDR-related foci, including those of γ-H2AX/53BP1, MRN (Mre11, Rad50, Nbs1 complex), FancD2 and Rad51 [20]. While γ-H2AX/53BP1 and FancD2 foci persist, Rad51 and Mre11 foci are strikingly absent following PML depletion (Fig. 2d). This suggests a connection of PML with a subset of DDR signaling pathways. Intriguingly, the affected DDR factors coincide with those shown to colocalize with PML [20].

**Figure 3:**
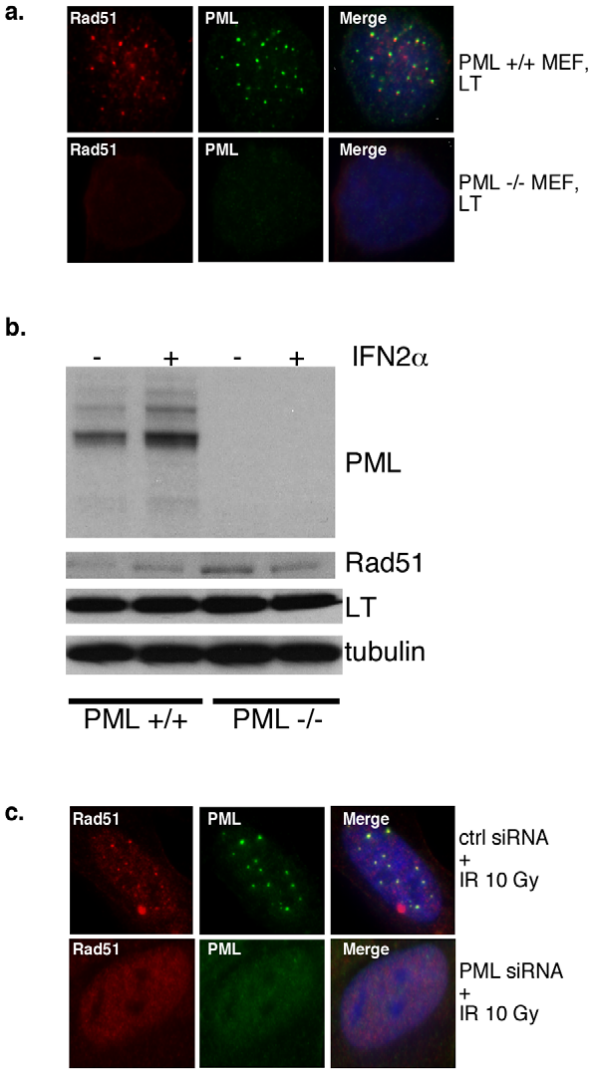
PML is required for Rad51 foci formation following LT expression in MEFs or following IR in normal human fibroblasts. **A**. PML-/- or +/+ MEFs expressing LT were subjected to immunofluorescence for Rad51 and PML. **B**. PML +/+ or -/- MEFs stably expressing LT were treated, or not, with interferon 2α (IFN-2α) to increase the PML expression to detectable levels. Cells were extracted and immunoblotted for PML, Rad51, LT and tubulin. Rad51 levels are not diminished in the PML null background. **C**. BJ/tert cells, transfected with control or PML siRNA, were exposed to 10 Gy of IR, followed 4 h later by immunofluorescent staining for Rad51 and PML.

We also incorporated PML knockout mouse embryo fibroblasts (MEFs) and their matched normal counterparts [9]. PML -/- and +/+ MEFs stably expressing LT were derived after retroviral transduction. Strikingly, wild-type MEFs expressing LT exhibit abundant Rad51 foci that are mostly colocalized with PML, whereas Rad51 is diffusely nuclear in PML null cells expressing LT (Fig. 3a). As confirmed by Western blotting, the PML deficient cells produced no PML signal even after interferon 2α treatment (Fig. 3b). Notably, expression of LT or Rad51 is equivalent in the presence or absence of PML. Thus, in this system, lack of Rad51 foci cannot be simply a consequence of reduced expression levels. Taken together, PML is a prerequisite for LT-induced Rad51 foci.

Since LT induces DNA damage [20], [31], including DSBs, we wondered if our observations reflect a general requirement of PML for proper localization of Rad51 at repair foci following DNA damage. To determine that, we transfected normal BJ/tert human fibroblasts with either control or PML siRNA, followed by 10 Gy of IR. In non-irradiated cells, Rad51 is located diffusely in the nucleoplasm and PML in foci, whereas 4 h after IR Rad51 is immobilized into foci that largely colocalize with PML (Fig. 3c and data not shown). However, PML knockdown causes an efficient elimination of Rad51 foci after IR and 4 h recovery (Fig. 3c). In conclusion, PML is required for focal accumulation of Rad51 after either LT expression or external DNA damage from IR, conceptually similar to the role of PML for TopBP1 and BLM localization in foci [12], [13].

### PML facilitates homology-directed repair following double-strand break induction

**Figure 4:**
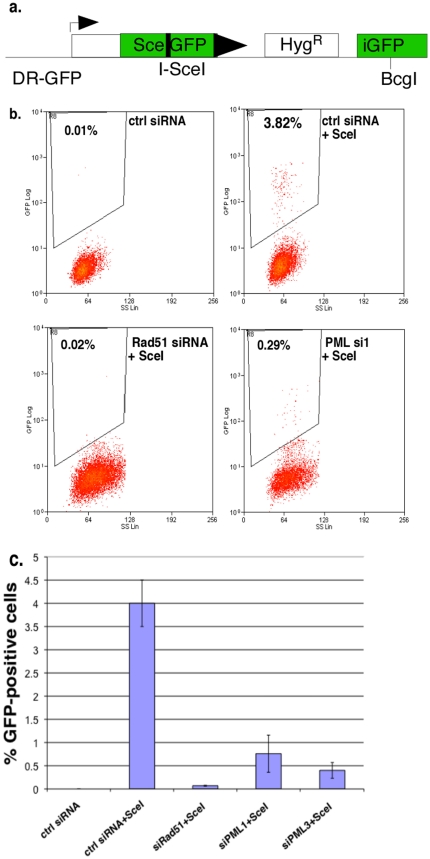
PML knockdown impairs homology-mediated repair. **A**. Schematic outline of the integrated DR-GFP reporter composed of two defective GFP genes that can be repaired by DSB-induced HR, resulting in GFP fluorescence. **B**. DR-U2OS were transfected with control, Rad51 or PML siRNA for 24 h, followed by transfection of I-SceI plasmid, or an empty vector, for another 48 h. Percentages of GFP positive cells arising from HR were determined by flow cytometry. A representative experiment is shown. **C**. Graph showing average number of GFP positive cells and SD from 3 independent experiments. Two independent PML siRNAs are included (si1 and si3).

Rad51 immunofluorescence analysis suggests that PML may be a prerequisite for efficient HR repair by localizing and stabilizing Rad51. To further investigate this possibility, we used DR-U2OS cells that harbor a single chromosomally integrated HR reporter construct (DR-GFP) used in several previous studies [32], [33], [34] (Fig. 4a). This reporter construct contains as direct repeats two independent, differentially mutated copies of a GFP gene, separated by a hygromycin resistance gene used for selection. The upstream copy of the GFP gene (SceGFP) has been modified to include a cleavage site for the rare-cutting endonuclease I-SceI and two in frame stop codons, whereas the downstream copy contains only an internal fragment of the GFP gene (iGFP). Upon expression of I-SceI and generation of a DSB, homology-mediated repair restores GFP expression by HR between the two mutated, non-functional GFP genes. GFP-positive cells are easily quantified by flow cytometry.

To investigate the role of PML in HR repair, we transfected DR-U2OS cells with control, Rad51 or PML siRNA for 24 h before expression of I-SceI for another 48 h. As shown in Fig. 4b, for control siRNA transfected DR-U2OS the spontaneous rate of HR repair is very low (0.01%), but highly elevated by I-SceI expression (3.82%). As expected, silencing of the essential recombinase Rad51 leads to a dramatic reduction in HR repair, even though I-SceI is expressed (0.02%). Strikingly, we found that siRNA-mediated depletion of PML causes a significant impairment of homology-mediated repair following I-SceI expression (0.29%)(Fig. 4b). The average percentages of GFP-positive cells from 3 independent experiments, including 2 independent PML siRNA sequences, are depicted in Fig. 4c. As a control, expression of a heterologous GFP plasmid (NZE-GFP), driven by the same promoter, is not significantly affected by the siRNA knockdowns (Fig. S2). Taken together, our results implicate PML in both Rad51 focal accumulation as well as efficient HR repair of chromosomal DSBs, although the deficiency upon PML loss is less severe than with Rad51 knockdown.

### PML is involved in processing of double-strand breaks and focal accumulation of Mre11, BRCA1 and RPA

**Figure 5:**
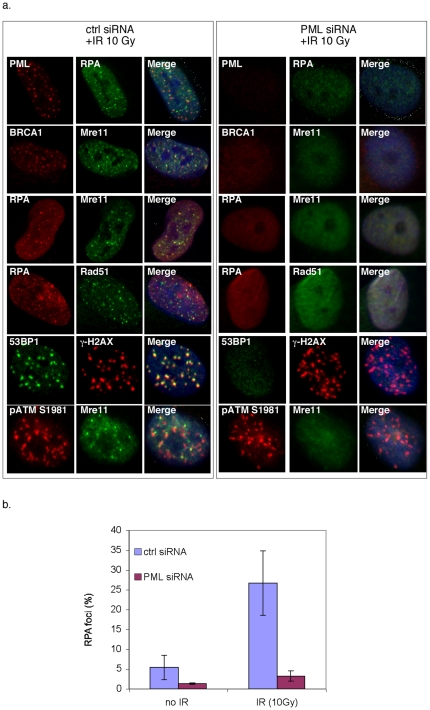
PML is required for irradiation-induced foci of Mre11, BRCA1 and RPA. **A**. BJ/tert cells transfected with control or PML siRNA were irradiated and 1.5 h later processed for immunofluorescence using PML, RPA, BRCA1, Mre11, Rad51, 53BP1, γ-H2AX or pATM S1981 antibodies in the combinations indicated. **B**. The frequency of cells with RPA foci was quantitated with or without IR in control or PML siRNA transfected cells.

Resection of DSBs is a fundamental prerequisite for Rad51 recruitment into foci and homology-directed repair [35]. Since the MRN complex and BRCA1 are implicated in resection, we analyzed their focal recruitment. As shown in Fig. 5a, when BJ/tert cells were transfected with a control siRNA, Rad51, Mre11 and BRCA1 are immobilized into foci 1.5 h after IR. Mre11 partially colocalizes with pATM S1981, which is believed to mark DSBs. When PML is depleted, Rad51, Mre11, BRCA1 and 53BP1 foci are strikingly absent after IR, while pATM S1981 and γ-H2AX foci persist (Fig. 5a). This suggests that the initial stages of ATM signaling proceed normally. To confirm this apparent defect in resection, we directly analyzed RPA foci. After end resection, the ssDNA is coated with RPA [23], [35]. As expected, a significant proportion of the irradiated cells transfected with control siRNA accumulate distinctive RPA foci that are colocalized with Rad51, Mre11 and PML (Fig. 5a). In striking contrast, PML knockdown cells are deficient for RPA foci formation, suggesting a failure in processing of DSBs. Repeated attempts to determine if CtIP accumulates in foci were unsuccessful, because of lack of a suitable antibody. The frequency of RPA foci in the presence or absence of IR was quantitated and is graphically depicted in Fig. 5b.

**Figure 6:**
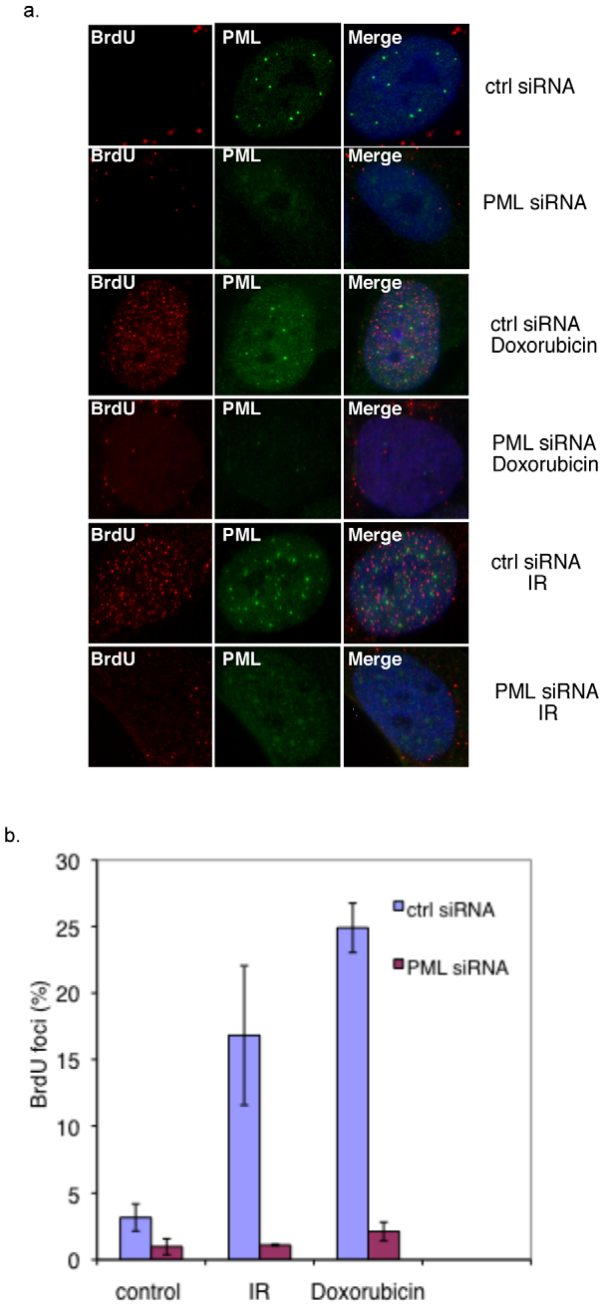
PML is required for ssDNA formation upon DNA damage. **A**. BJ/tert cells transfected with control or PML siRNA were labeled with 10 µg/ml BrdU for 4 h prior to DNA damage. Cells were non-treated or exposed to 10 Gy of IR for 1 h or 0.25 µg/ml doxorubicin for 4 h. Immunofluorescent detection of incorporated BrdU without denaturation and PML was performed. **B**. Quantitation of cells with BrdU foci in control or PML siRNA transfected BJ/tert cells with or without DNA damage.

The failure to generate RPA foci could result from either absence of ssDNA or a defect in loading of RPA. To distinguish between these possibilities, we directly monitored presence of ssDNA by incorporation with BrdU followed by immunofluorescent detection without denaturation. This technique was previously demonstrated to allow detection of ssDNA foci [13], [24], [36]. As shown in Fig. 6a, BrdU foci are largely absent in control or PML siRNA transfected BJ/tert cells. However, upon IR, or doxorubicin treatment, BrdU foci accumulate in a substantial fraction of cells transfected with control siRNA. Most BrdU foci were not colocalized with PML, at least at these time points. Strikingly, BrdU foci were absent when PML was depleted with specific siRNA (Fig. 6a). Quantitation of cells with BrdU foci under the various conditions is outlined in the graph of Fig. 6b. Taken together, these observations strongly suggest that PML is involved in resection or another DNA processing event that generates ssDNA.

### PML can associate with the Rad51 recombinase

**Figure 7:**
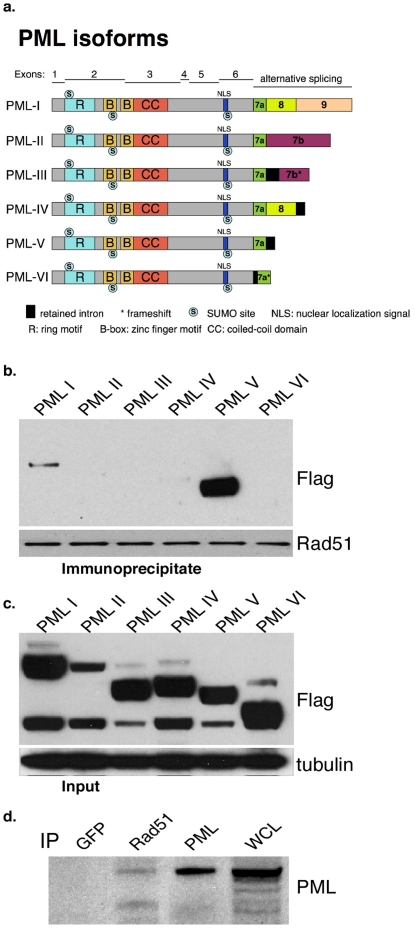
PML can associate with Rad51. **A**. Schematic drawing of the various PML isoforms generated by differential C-terminal splicing. PML features such as the ring motif, B-box, coiled-coil domain, nuclear localization signal and SUMOylation sites are indicated. **B**. Lysates from U2OS/T cells transfected with flag-tagged PML I to VI expression vectors were immunoprecipitated for endogenous Rad51 with rabbit polyclonal antibody, followed by flag immunoblotting for coprecipitating PML. The blot was reprobed for Rad51 to show equal immunoprecipitation. **C**. Expression of the Flag-tagged PML isoforms was determined by Western blotting with Flag antibody. The blot was also probed for tubulin to show equal loading. **D**. BJ/tert LT cell lysates were immunoprecipitated with GFP (negative control), Rad51 or PML antibody, followed by PML immunoblotting to demonstrate endogenous complex formation. A whole cell lysate (WCL) was included.

Given the functional link between PML and Rad51, we hypothesized that they might be in a complex together. Since there are multiple PML isoforms due to differential splicing (schematically depicted in Fig. 7a), and we sought to determine which ones are potentially binding competent, we transfected Flag-tagged expression vectors for each isoform into U2OS/T cells [37]. U2OS/T cells were chosen, because they are highly transfectable, and endogenously expressed LT can induce DDR activation [31]. After immunoprecipitation of endogenous Rad51, Western blotting clearly demonstrates specific coprecipitation of PML V, as well as PML I to a much lesser extent (Fig. 7b). Immunoblotting of the lysates with Flag antibody shows approximately equal expression of the various isoforms (Fig. 7c). The preference for isoform V is intriguing, as not much is known about this species other than its slow turnover at PML NBs and it being a target of the adenovirus E1B 55k protein [38], [39]. While there is no significant interaction between PML isoforms and Rad51 in U2OS cells, treatment with a DNA damaging agent led to a similar, but not identical, interaction pattern as in U2OS/T (Fig. S3). We also examined interaction between endogenous PML and Rad51. Lysates from BJ/tert LT cells were immunoprecipitated for GFP (negative control), Rad51 or PML, followed by Western blotting with PML antibody to detect complexes. As shown in Fig. 7d, specific coimmunoprecipitation can be detected between Rad51 and PML, thus confirming the interaction with the endogenous proteins.

### PML is required for Chk1 activation

**Figure 8:**
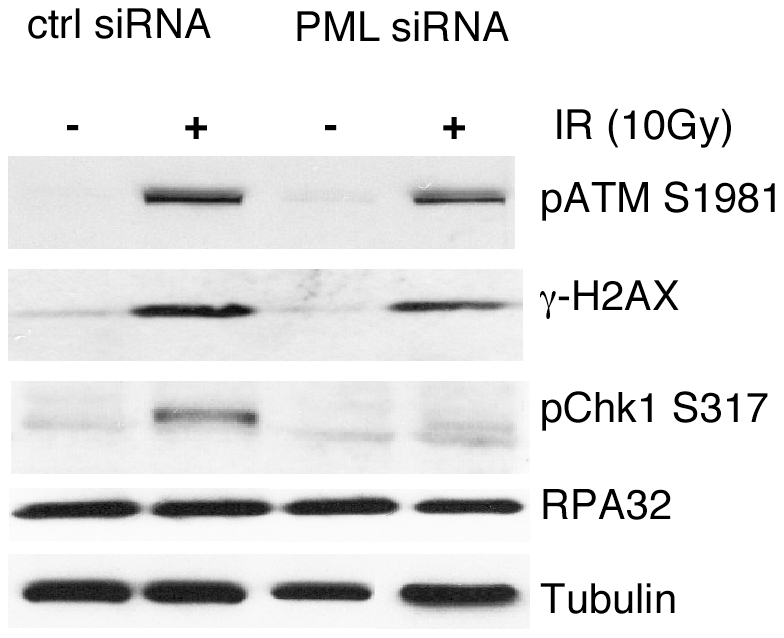
PML is required for activation of Chk1. BJ/tert cells were transfected with control or PML siRNA, followed by 10 Gy of IR and 4 h recovery. Lysates were analyzed by Western blotting for pATM S1981, γ-H2AX, pChk1 S317, RPA32 and tubulin.

Since PML deficiency causes a failure for RPA to enter into foci, it might be expected that ATR/ATRIP cannot be recruited to ssDNA, and Chk1 activation would be compromised [40]. When BJ/tert cells are transfected with control siRNA, activated pChk1 S317 can be detected after IR, but this response is attenuated upon PML knockdown (Fig. 8). However, accumulation of pATM S1981 and γ-H2AX following IR are not greatly affected by PML depletion. Moreover, despite a failure to form RPA foci without PML, RPA levels are unchanged.

## Discussion

In this study, we used the SV40 LT viral oncoprotein model to elucidate novel connections between PML and the HR machinery. A significant conclusion is that PML is critical for proper localization of Rad51 in nuclear foci, as well as for efficient homology-directed repair. We found that in LT-expressing cells, Rad51 foci depend on PML. Although the emphasis here was on Rad51, PML clearly plays a role in localizing, or otherwise regulating, additional DDR factors such as TopBP1, BLM, MRN, RPA and BRCA1 ([12], [13], Fig. 5a). The relative significance of each of these interplays remains to be established.

LT was previously found to induce DDR and repair pathways, the evidence including prominent foci of Rad51, which are believed to reflect genuine sites of ongoing or completed HR repair [20], [24], [25], [26], [27]. Importantly, our observations could be generalized beyond LT to DSBs inflicted by IR, where Rad51 foci formation also depends on PML. Using a previously established and characterized GFP-based assay for homology-directed repair [32], [33], [34], we show that PML depleted cells exhibit a substantial defect following an I-SceI-induced break. These observations implicate PML NBs in control of homology-directed DSB repair, an important tumor suppressor mechanism believed to be essential for BRCA1 and BRCA2 function to prevent breast, and other, cancers [32], [41].

Several connections between PML NBs and DNA repair have been made prior to this study. The MRN complex, p53 and γ-H2AX were found to colocalize in foci also containing PML after DNA damage [14]. BLM was found to colocalize with PML NBs and RPA in Rad51 foci during late S/G2 and in response to DNA damage [13]. However, in PML deficient cells or APL blasts, BLM is delocalized. BLM, together with PML and Rad51, were also found to accumulate at sites of ssDNA after IR, thus delineating sites of presumptive DNA repair [13]. Furthermore, PML is a critical component of APBs (ALT-associated PML bodies) together with the MRN complex, RPA, Rad51 and telomere binding proteins (TRF1/TRF2) in the context of the alternative lengthening of telomeres (ALT) pathway responsible for telomere maintenance [42]. PML was also found to colocalize with and stabilize the DDR protein TopBP1 following IR [12].

The exact role that PML plays in orchestrating repair is not clear but may involve the ability of PML NBs to act as DNA damage sensors or processing sites [3], [11], [36]. Notably, ssDNA is found at PML NBs as early as 1 h after UV [36] or IR [12], but ssDNA foci disappear when PML is depleted ([36], Fig. 6). Moreover, ssDNA accumulates together with PML in viral replication centers following polyomavirus infection [18]. PML NBs are not generated *de novo* at sites of DNA damage; rather it appears that ssDNA is recruited to existing PML NBs [36]. Given the repair connection, PML NBs can be compared to irradiation-induced foci (IRIF), but unlike these transient foci that assemble following DNA damage, the PML NBs are permanent structures present in most cell types and throughout the cell cycle. Interestingly, there is also evidence that PML NBs act as sensors of DNA damage, since they increase in number by a fission mechanism dependent on ATM/ATR signaling [3], [11].

Our current observations add important insight. Since RPA, the MRN complex and BRCA1 fail to be recruited into foci upon DNA damage, when PML is depleted, this suggests a role of PML in coordinating DSB processing, a prerequisite for Rad51 foci and repair [23], [35]. Indeed, our observations indicate that PML is required for ssDNA generation following DSBs (Fig. 6), which confirms and extends previous observations in UV-irradiated cells [36]. The exact role that PML plays in DNA processing remains to be shown. Moreover, it is not clear if failure to generate ssDNA explains all of the observed defects, such as Rad51, Mre11, BRCA1 focal accumulation, homology-directed repair and SV40 replication in vivo.

Similarly to the studies on BLM and TopBP1 [12], [13], we find that PML is also required for Rad51 accumulation and localization in foci. Although we found PML to be necessary for maintaining the steady state levels of Rad51 in U2OS and BJ/tert cells, and Rad51 levels may sometimes determine its ability to form nuclear foci, we do not believe the decrease in Rad51 expression fully explains the absence of foci. Thus, decreased levels of Rad51 do not necessarily preclude foci formation, and BRCA2 deficient cells fail to exhibit Rad51 foci without a change in Rad51 expression [25]. Most importantly, in the PML knockout MEFs, Rad51 levels are not decreased, yet foci formation is abrogated.

How is PML recruiting Rad51 to sites of DNA damage? Our results indicate that PML and Rad51 can interact as detected by coimmunoprecipitation. However, it is not clear, whether this is direct or mediated by additional factors like BLM, BRCA2 or DAXX. A potential mode of recruitment involves the SUMO interaction motif in Rad51, which might make contacts via the three SUMO modification sites on PML [43]. The SUMO interaction motif in BLM is required for its recruitment to PML NBs and SUMO modification [44], [45]. Interestingly, RPA70 is also SUMOylated, which is believed to facilitate localization of Rad51 to DNA damage foci by direct recruitment [46]. Future investigations will reveal the exact mechanisms whereby Rad51 is recruited to PML NBs, and whether it depends on SUMO modification or non-covalent interaction.

PML, like BRCA2, appears to be physically and functionally linked with Rad51 [47]. While total loss of HR repair likely is lethal, as suggested with Rad51 mouse knockouts [48], PML is not required for cell viability [9]. PML deficient cells are unlikely to be completely defective in homology-directed repair, as suggested by the low, but above background, frequency of GFP-positive cells, thus suggesting that PML facilitates the repair process.

Previous studies implicated PML in genome stability control, since spontaneous sister chromatid exchanges (SCEs) were elevated in mouse PML knockout fibroblasts [10]. Because SCEs appear to depend on Rad51 for HR [49], it seems contradictory to our observations that Rad51 functions depend on PML following DNA damage. However, there are several distinct HR repair pathways that depend on Rad51, and it may be that PML only contributes to a subset. Also, it is possible that spontaneous SCEs differ mechanistically from those induced by DNA damage. Interestingly, a similar phenomenon has previously been observed in FANCC and Rad18 knockout cells [50].

Here, we also characterized the intricate relationship between SV40 and PML NBs. Previous reports had demonstrated that SV40 genomes are localized adjacent to PML NBs, and it was suggested that efficient SV40 replication is associated with specific nuclear sites proximal to PML NBs [16], [17], [19]. However, several viruses have in fact been shown to disrupt PML NBs, in part to abrogate the anti-viral interferon response [2], [4]. Our results are consistent with a unique positive role of PML NBs in SV40 replication, as it was originally inferred. It was not known how PML might mechanistically contribute to facilitate SV40 replication. We show that the virus likely targets PML NBs early in infection to gain access to DNA repair factors. Notably, the Rad51 recombinase is required for efficient viral replication [20], [51]. Since knockdown of the CtIP factor required for resection also impairs origin-dependent replication, it suggests that the PML contribution in replication may also be due to involvement in HR processes.

How is LT targeted to PML NBs? While the initial report indicated that LT expression, as well as the core origin or replication, were both required for targeting to PML NBs [19], our subsequent study utilizing a pre-extraction step in immunofluorescence found LT to reside on chromatin at, or near, PML NBs, in the absence of an origin [20]. It remains to be shown if LT resides at PML NBs, because of sumoylation or non-covalent SUMO interactions. The previously reported activation of a DDR by SV40 infection, and by LT alone, is likely to play a role [20], [52], [53]. Following infection, key components of the DDR, like ATM, RPA, ATRIP, 53BP1, γ-H2AX are recruited into viral replication centers, with PML NBs juxtaposed to these. In a striking parallel, PML and DNA repair proteins are recruited to both viral replication compartments and sites of DNA damage, thus making it possible that viral genomes, and LT itself, are recruited to PML NBs as part of a DDR [15]. A mutant of LT in the Bub1 binding site fails to induce γ-H2AX/53BP1 foci, a hallmark of the DDR, and is delocalized from PML NBs, consistent with a potential link [20], [31]. Taken together, the positive regulatory role of the DDR in SV40 replication may in part involve localization near PML NBs.

Although we observed a clear and consistent inhibition of SV40 replication in permissive cells following depletion of PML using 3 independent siRNA sequences, a previous study had found no effect [18]. However, our conclusion is further substantiated by identical results in human U2OS/T cells and in COS-1 cells using an alternative replication assay based on a luciferase reporter. To reconcile this discrepancy, we tested the same target sequence that was used before (si5, [18]). In our hands, this particular siRNA sequence did not efficiently knock down PML expression, and consequently, replication was not greatly affected (Fig. 1d). Alternatively, these contrasting results could also be attributed to cell type differences (MCF-7 versus COS-1 or U2OS/T), replication assay techniques (real-time PCR versus Southern blotting), selected time points or use of full genome versus the minimal origin of replication.

In conclusion, the SV40 LT model has been used in a novel way to decipher links between PML, DNA repair and viral replication activities. We have discovered that PML appears to play a key role in coordinating or integrating repair functions. The exact mechanism for this is unknown but may involve a role of PML NBs in orchestrating a microenvironment, where regulated recruitment and release of repair factors can occur. An emerging concept in cancer therapeutics is the synthetic lethality-based treatment of tumors harboring BRCA1/2 mutations with poly(ADP-ribose) polymerase (PARP) inhibitors [54], [55]. Since it appears that this strategy might apply to other cancers characterized by HR deficiency, it is of great interest to investigate if tumors with dysfunctional PML are susceptible to this treatment regimen [56].

## Materials and Methods

### Cell culture

U2OS, U2OS/T, DR-U2OS (kindly provided by Maria Jasin), PML+/+ and PML-/- MEFs [9], COS-1 and Phoenix amphotropic cells were grown at 37°C in 5% CO_2_ using Dulbecco's modified Eagle's medium (DMEM) (Lonza) supplemented with 10% fetal calf serum (FCS) (Hyclone) and 1% penicillin/streptomycin. BJ/tert and BJ/tert LT were cultured in 80% DMEM/20% Medium 199 (Invitrogen) supplemented with 10% FCS (Hyclone) and 1% penicillin/streptomycin.

### Plasmids and transfection

Expression vectors (pCI-Neo, Promega) for PML isoforms I to VI were kindly provided by Keith Leppard [37]. A retroviral vector for shRNA-mediated PML knockdown (pSiren RetroQ, Clontech) was generously provided by Thomas Stamminger [29]. The retroviral pLBNCX LT vector has been previously described, as was the SV40 origin-containing plasmid pSV01ΔEP [20]. The plasmids pCbA SceI and control NZE-GFP were obtained from Maria Jasin. Cells were transfected with Fugene-6 according to the manufacturer's protocol (Roche).

### siRNA-mediated knockdown

Five individual siRNAs were custom synthesized for PML (Dharmacon) based on the following cDNA sequences: si1 5′ AGATGCAGCTGTATCCAAG 3′ [29]; si2 5′ GAGTCGGCCGACTTCTGGT 3′ [57]; si3 5′ ATGGCTTCGACGAGTTCAA 3′; si4 5′ AACCACAGCCCAGAAGAGG 3′ and si5 5′ GCATCTACTGCCGAGGATG 3′ [18]. Si1-3 and si5 were designed against the human PML sequence, and targeted the monkey counterpart with variable efficiency, whereas si4 was made against monkey PML. Si2 [57] and si5 [18] were found to be inefficient for PML depletion. All siRNAs were targeted against the sequence conserved in all major isoforms (I to VI). Rad51 or CtIP knockdown was performed using a SMARTpool (Dharmacon). A non-targeting siRNA (Dharmacon) was used for negative control.

### Viral infections

Retroviral packaging was performed by transfection of Phoenix amphotropic cells with vector DNA, followed 48 h later by filtering of the medium through a 0.45 µm membrane (Millipore). Retrovirus was stored at −80°C in aliquots. Cells were infected overnight with retrovirus in the presence of 8 µg/ml of polybrene. Selection with blasticidin (5 µg/ml) or puromycin (1.5 µg/ml) was started 48 hr post infection.

### HR reporter assay

The efficiency of HR was evaluated using a GFP-based reporter assay that has been previously described [32], [33], [34]. We used the DR-U2OS cell line containing a mutant (nonfunctional) GFP sequence with an 18 bp I-SceI recognition site. DSBs were introduced into an integrated GFP gene using the I-SceI endonuclease, and repair of these breaks by HR resulted in expression of a functional GFP gene, which was quantitated by measuring GFP signal using flow cytometry. DR-GFP cells were transfected with control, Rad51 or PML siRNA for 24 h, followed by transfection of I-SceI expression vector for another 48 h. Cells were processed for flow cytometry on a Dako MoFlo machine. GFP expression in gated live populations was analyzed using Summit 4.3 Software.

### Antibodies

Primary mouse monoclonal antibodies were purchased from MBL (PML clone 1B9), Millipore (Rad51 clone 3C10, PML clone 36.1-004, γ-H2AX clone JBW301), BD Biosciences (BrdU, clone B44), Santa Cruz (PML clone PG-M3), Sigma (α-tubulin clone B-5-1-2, Flag M2, vinculin clone hVin-1), Rockland (pATM S1981) and Neomarkers (RPA). Monoclonal antibodies to PML (5E10) and CtIP (14-1) were kindly provided by Roel van Driel and Richard Baer, respectively. Monoclonal antibodies against LT (PAb416 and PAb423) were previously described [20]. Primary rabbit polyclonal antibodies were purchased from Novus Biologicals (Mre11 (100–142), Nbs1 (100–143)), R&D Systems (pChk1 S317), Santa Cruz (Rad51 (H-92), SV40 LT (V-300) and PML (H-238)) and Bethyl laboratories (RPA). Secondary antibodies for immunofluorescence staining were obtained from Invitrogen (Alexa 488-labeled) and Jackson ImmunoResearch (Cy3-labeled).

Interferon 2α (IFN-2α) was purchased from PBL interferon source.

### Immunoprecipitation

Cells were washed with ice cold PBS and scraped into (high salt) lysis buffer I [25 mM HEPES pH 7.0, 0.5 M NaCl, 0.1% Nonidet P-40, 1 mM sodium butyrate] supplemented with protease and phosphatase inhibitors [37]. Extraction was continued 20 min on ice, followed by sonication and centrifugation at 12,000 xg for 20 min. The lysate was mixed with an equal volume of (low salt) lysis buffer II [25 mM HEPES pH 7.0, 0.05 M NaCl, 1 mM sodium butyrate] also supplemented with protease/phosphatase inhibitors. Antibody was added to the lysate and the mixture rotated at 4°C for 2 h, followed by addition of pre-washed protein A/G agarose beads (Santa Cruz) and incubation another 45 min. Beads were then washed 6 times with wash buffer [50 mM Tris-HCl pH 7.4, 150 mM NaCl], boiled with Laemmli sample buffer and loaded on a 4–12% NuPage gel (Invitrogen).

### Immunofluorescence

Cells were seeded on glass coverslips coated with poly-L-lysine (Sigma) and allowed to attach for 48 h. After washing with PBS, cells were fixed in 4% paraformaldehyde in PBS for 15 min at 4°C. The fixed cells were washed twice in PBS and permeabilized with 0.5% Triton X-100 in PBS for 5 min. After 30 min of blocking with 10% normal goat serum in PBS, cells were washed in PBS and incubated with primary antibody. After three 5 min washes with PBS, cells were incubated with secondary antibody, followed by another three PBS washes. After brief DAPI staining, the cover slips were mounted on glass slides and cells visualized on an Olympus fluorescence microscope. Images were captured using a Spot advanced imaging system.

### Viral replication assays

Viral replication assays were performed as previously described [20], [58]. Briefly, COS-1 cells were transfected with siRNAs at a final concentration of 40 nM by Lipofectamine RNAiMAX (Invitrogen). After 48 h transfection of siRNAs, pSV01ΔEP was transfected by Fugene 6 (Roche) for another 24 h. Cells were then lysed with RIPA buffer [50 mM Tris pH 8.0, 150 mM NaCl, 1% Nonidet P-40, 0.5% sodium deoxycholate, 0.1% SDS] for Western blot analysis or lysed to prepare low molecular weight DNA according to the Hirt protocol. Low molecular weight DNA was digested with DpnI/HincII, resolved on a 0.8% agarose gel and transferred to Hybond-N nylon membrane (GE Healthcare Life Sciences). The membrane was then UV cross-linked and hybridized at 42°C overnight with ^32^P-labeled 314 bp EcoRI restriction fragment of pSV01ΔEP. After multiple washes, the membrane was exposed to X-ray film.

### Luciferase assays

COS-1 cells were plated 24 h before transfection in a 24-well plate at a density of 35,000 cells per well. siRNAs were tranfected into COS-1 cells at a final concentration of 40 nM by Lipofectamine RNAiMAX (Invitrogen). After 48 h transfection of siRNAs, the luciferase DNA replication assay was performed as described [21]. Briefly, 17.5 ng of SV40 origin-containing plasmid pFLORI40 and 3.5 ng of pRL were transfected by Fugene 6 (Roche) for another 24 h. Firefly and Renilla luciferase activities were measured using the Dual-Glo Luciferase assay system (Promega) in a Synergy 2 Multi-Mode Microplate Reader (Biotek Instrument).

## Supporting Information

Figure S1 PML knockdown reduces viral replication in COS-1 cells as measured by a luciferase reporter assay. (a) Schematic of the two reporter constructs. The firefly luciferase gene is linked to an SV40 origin in pFLORI40, whereas the Renilla luciferase is serving as an internal control in pRL. (b) COS-1 cells were transfected with siRNA for 48 h, followed by transfection of pFLORI40 and pRL for another 24 h. Average values of firefly luciferase (Fluc) activity relative to Renilla luciferase (Rluc), together with standard deviation, are shown for triplicate samples.
(TIF)

Figure S2 Various siRNAs do not significantly affect expression from a control GFP reporter. U2OS were transfected with siRNA for 24 h, followed by transfection of the control plasmid NZE-GFP for another 48 h. GFP expression was evaluated by gating the proper populations using flow cytometry.
(TIF)

Figure S3 Interaction of Rad51 with PML appears to be induced by DNA damage. (a) U2OS cells were transfected with flag-tagged PML I-VI expression vectors, followed 48 h later by immunoprecipitation of endogenous Rad51 and blotting for flag. (b) Same as in (a), except U2OS cells were treated with 1 mM hydroxyurea (HU) for 3 h, followed by 8 h recovery.
(TIF)
