# Supplementary figures and images for: Functional Connection between Rad51 and PML in Homology-Directed Repair
Source: PLoS One. 2011 Oct 5;6(10):e25814. doi: 10.1371/journal.pone.0025814 (PMC3187806; doi:10.1371/journal.pone.0025814)

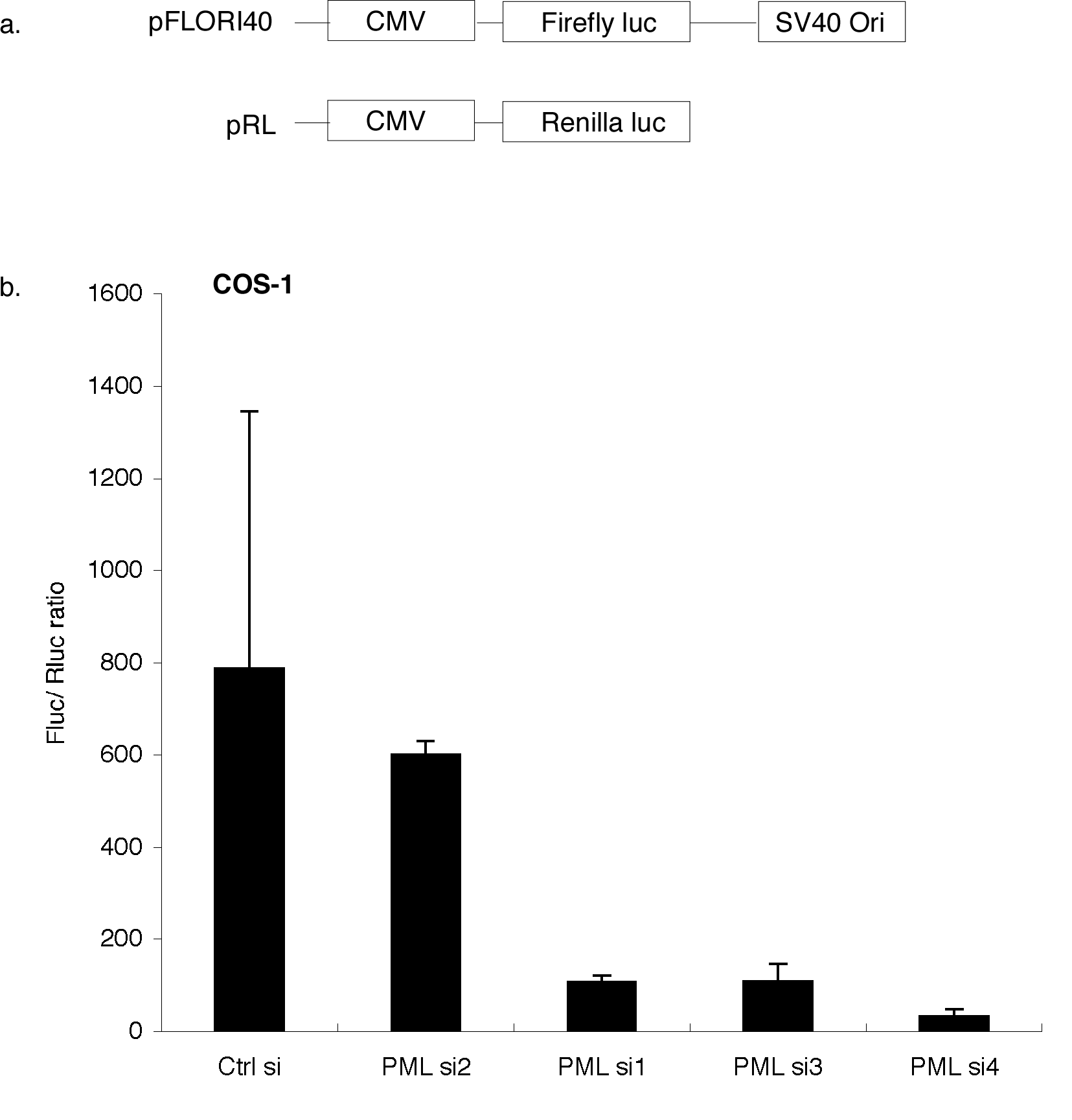

Supplement: Figure S1 — PML knockdown reduces viral replication in COS-1 cells as measured by a luciferase reporter assay. (a) Schematic of the two reporter constructs. The firefly luciferase gene is linked to an SV40 origin in pFLORI40, whereas the Renilla luciferase is serving as an internal control in pRL. (b) COS-1 cells were transfected with siRNA for 48 h, followed by transfection of pFLORI40 and pRL for another 24 h. Average values of firefly luciferase (Fluc) activity relative to Renilla luciferase (Rluc), together with standard deviation, are shown for triplicate samples. (TIF) [file pone.0025814.s001.tif]

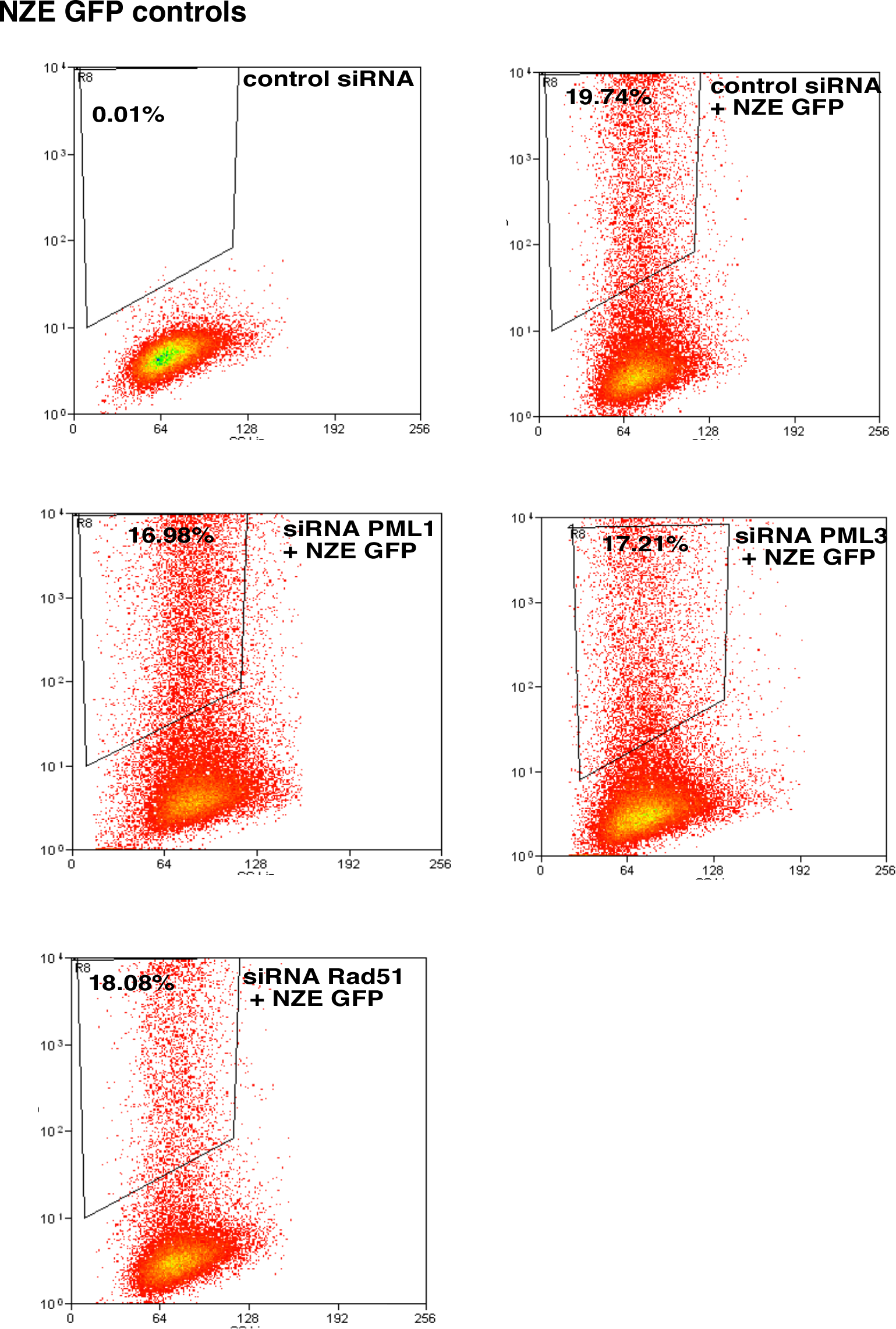

Supplement: Figure S2 — Various siRNAs do not significantly affect expression from a control GFP reporter. U2OS were transfected with siRNA for 24 h, followed by transfection of the control plasmid NZE-GFP for another 48 h. GFP expression was evaluated by gating the proper populations using flow cytometry. (TIF) [file pone.0025814.s002.tif]

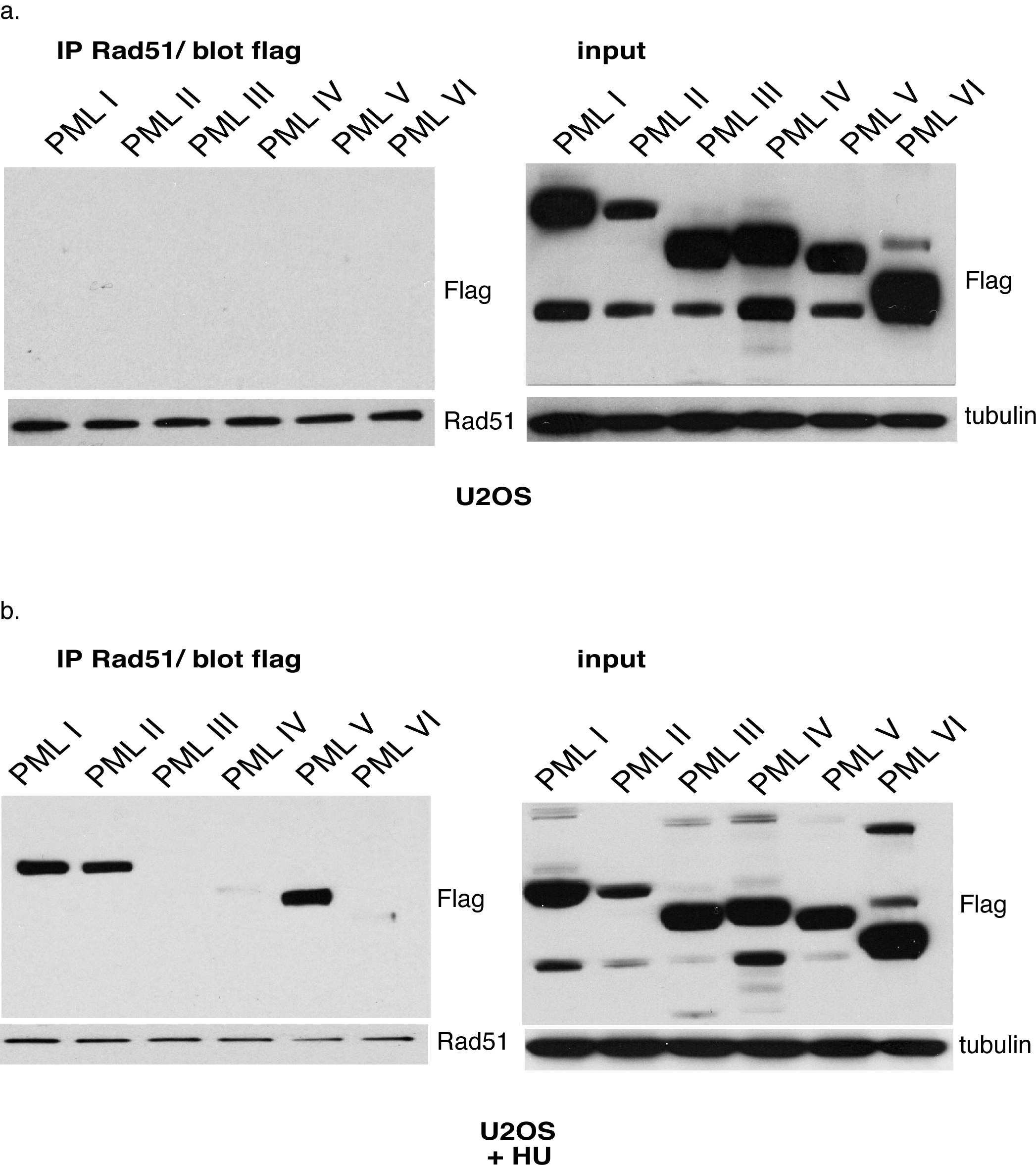

Supplement: Figure S3 — Interaction of Rad51 with PML appears to be induced by DNA damage. (a) U2OS cells were transfected with flag-tagged PML I-VI expression vectors, followed 48 h later by immunoprecipitation of endogenous Rad51 and blotting for flag. (b) Same as in (a), except U2OS cells were treated with 1 mM hydroxyurea (HU) for 3 h, followed by 8 h recovery. (TIF) [file pone.0025814.s003.tif]
